# Some Disorders of Adolescence

**Published:** 1887-01

**Authors:** John M. Keating

**Affiliations:** Philadelphia


### Some Disorders of Adolescence, Especially in Girls.

BY JOHN M. KEATING, M.D., OF PHILADELPHIA.

There is a large amount of literature on this subject, but it is mostly confined to monographs or articles appearing in popular magazines. These do undoubtedly a great deal of good, but at the same time it seems to me that there is a medical side of the question which is equally important.

We all recognize the very great importance of impressing upon mothers the value of all that tends toward muscular development in growing girls. They should be symmetrically developed, should have full chests, straight backs, and strong limbs. We should also urge the importance of clothing of light weight and loose fitting, the principal strain being on the shoulders, not on the waist and hips, and also the evil results of cramped, stooped positions in the school-room, eye strain, and bad ventilation. We all urge these matters daily, and we all know but little attention is paid to them. But there are certain forms of various disorders which occur about the time of the second dentition which deserve more than a passing notice. These are manifested either as a chorea, nervous excitement such as night terrors, and various mental disturbances misnamed hysteria, gastro-intestinal disorders, and evidences of mal-nutrition. The child will probably become languid, suffer with frontal headache, become peculiar in her disposition and show fits of temper, shun the society of other children, lose her appetite, become despondent, and possibly develop a local twitching of some of the facial muscles. It is customary to say that this is all reflex,* is possibly the warning that the system is undergoing some change preparatory to the menstrual functions—that it is in fact a true hysteria. This may or may not be the case. My own impression is that it is often due to the anæmia brought about by rapid growth and development, with faulty assimilation and deficient oxygenation. In my experience such cases present two types, the one essentially nervous, just described, the other the so-called strumous or lymphatic, in whom the want of proper assimilation is shown by a large amount of stored fat, and the anæmia by excessive pallor.

In the first case, the mother will tell you at once that her child cannot take iron, that she has frequent nose-bleeds, and that she feels confident that if iron could be given it would be of great service. The nervous system seems to run riot, but this very excitement in itself is an evidence of the demand on the part of nature for a blood supply which is nutritious and well oxygenated. All the exercise in the world, all the most nutritious and sustaining of foods, will have no effect, until the digestive organs are made to perform their normal functions. If you examine the tongue you will find it coated; the breath is heavy, the bowels

* Sufficient attention has not been called to the disturbances caused by the pressure of the twelfth year molars. These may show themselves in either dental neuralgia, or in fact, any form of trifacial neuralgia, gastric disorders, or mental peculiarities, amounting to melancholia or symptoms of acute meningeal irritation.

are sluggish, the appetite is perverted, the child craves extraordinary articles of food, especially acids and sweets. She has a disgust for her regular meals. There is flatulence, cardiac palpitations, asthma, after exertion. The urine is either scanty and high colored, or very copious and of low specific gravity. If the menses have been established they are scanty, colorless, and irregular, or there is a leucorrhœa. In these cases the recommendation of popular writers for gymnastics, friction, mild diet, etc., are admirable after the digestive organs have been cleared of their accumulation of ashes, and the normal functions whipped into activity. For an infant I have the greatest confidence in small doses of calomel, with soda bicarb., and ipecac, frequently repeated; but for the cases we now speak of, I much prefer the following:

| | | | |
|---|---|---|---|
| ℞ | Acid nitro-muriat. dil., | | ♏ xl. |
| | Succus. Tarax., | | ♍ lxxx. |
| | Vin. pepsini, | q. s. ad. | ℥j. |

Sig.— Teaspoonful in water after meals three times daily, with a half teaspoonful of the fluid extract cascara sagrada every night until the bowels become regular.

After taking these for a few days, if the tongue has become clean, the complexion clearer, the patient can be placed on the following, instead:

| | | | |
|---|---|---|---|
| ℞ | Hydrarg. chl. corros., | | gr. $\frac{1}{8}$. |
| | Liq. arsenici chlor., | | gtt. xij. |
| | Tinct. ferri chlor.., | | ʒj. |
| | Syr. limonis, | | ℥j. |
| | Aquæ, | q. s. ad. | f℥ vj. |

Sig.—Tablespoonful after meals, and the laxative continued, if necessary, at night.

As far as the general treatment is concerned, the little patient should be sponged every morning with tepid water, she should stand in a tub, and have a pitcher of it poured down her spine from the nape of her neck, and then be thoroughly rubbed with a soft Turkish towel into a glow. The breakfast should consist of milk warm, or cocoa, a soft-boiled egg, or rare pieces of steak or chop, either oatmeal, cracked wheat, grits, or Indian meal alternating; bread and butter, with hot cakes. For dinner soup, rare meat, fresh vegetables, and very little water. For supper, stewed fruits, bread and butter, warm milk or cocoa, with tea, not coffee. She should retire early, and not be permitted to read at night. The supply of oxygen should come from out-door exercise, not an over-indulgence in walks or games that fatigue; let the school hours be limited to the early part of the day, and avoid that abomination of preparing lessons in the afternoon or evening for the next day's recitations.

In about a week's time the girl will be able to bear the iron alone, and the tincture of the chloride can be given in ten or fifteen drop doses for some time, or a chalybeate water can be given with arsenic. The digestive organs will now also tolerate milk in large quantities, provided it is of medium richness, is fresh, and given warm.

But this is not all. There are very many cases of a highly nervous type, which, despite the most careful treatment, will not improve at home. The constant association with parents of like temperament, however solicitous they may be in carrying out instructions, is of itself a cause of nervous irritation.

It may be necessary to send such children from home, either to some relative, living possibly in the country or some distant city, or perhaps to some suburban or country boarding school, where a thorough change of air and scene, the association with girls of a different temperament, will work wonders.

For the strumous type, the same preparatory treatment may be instituted, and for such I would not hesitate to push the iron, phosphates, cod-liver oil as soon as possible. Change of air to the sea-shore is advisable. There is little trouble in the home treatment on these latter cases; there is rarely a conflict of authority in such families.

Although I have intimated that the ovaries have little to do with the production of these conditions, I feel satisfied that the weakness, constipation, faulty clothing, eye strain, or dental pressure, will eventually tend to the production of uterine derangements—anæmia being the cause, due to a deficient assimiliation, from digestive disturbances, want of fresh air and healthful exercise, reflex irritation, and afterwards uterine disorders follow—*post hoc* instead of *propter hoc.—Med. and Surg. Rep.*

